# Personal and family factors for emotional distress in adolescents with chronic disease

**DOI:** 10.3389/fpsyg.2023.1304683

**Published:** 2024-01-08

**Authors:** Silvia Postigo-Zegarra, Konstanze Schoeps, Marián Pérez-Marín, Laura Lacomba-Trejo, Selene Valero-Moreno

**Affiliations:** ^1^Personality, Assessment and Psychological Department, Faculty of Psychology and Speech Therapy, University of Valencia, Valencia, Spain; ^2^Faculty of Health Sciences, European University of Valencia, Valencia, Spain; ^3^Educational and Developmental Psychology Department, Faculty of Psychology and Speech Therapy, University of Valencia, Valencia, Spain

**Keywords:** pediatric chronic illness, anxiety, depression, illness threat perception, self-esteem, family functioning

## Abstract

**Introduction:**

Physical and psychological comorbidity is a challenge for public health, especially in the adolescent stage due to the difficulties of this age. The salutogenic perspective emphasizes general psychological resources such as self-esteem but also highlights the role of contextual factors such as family members in promoting health. From this perspective, the objective of this study was to analyze the joint influence of demographic factors (sex, age and type of chronic disease), personal factors (self-esteem and perception of threat of the disease) and family factors (affection and communication, psychological and behavioral control) of risk of emotional distress (anxiety-depressive symptoms) in chronically ill adolescents.

**Methods:**

The study is a cross-sectional design with 495 adolescents with chronic disease aged 12–16 years. In order to obtain the results, a linear methodology was used to compare means and perform regressions to predict belonging to the anxiety and depression typologies. Four typologies were constructed: typology I (high anxiety and depression scores); typology II (high anxiety and low depression scores); typology III (low anxiety and high depression scores) and typology IV (low anxiety and depression scores).

**Results:**

The results were consistent with the salutugenic theory. Potential mediating or moderating roles of age, sex, self-esteem, perceived threat and psychological and behavioral control marked the differences between the typologies.

**Discussion:**

This population could benefit from interventions focused on family flexibility.

## 1 Introduction

A recent systematic review indicated that the onset of the first mental disorder usually occurs before the age of 14 and that the average age of confirmed mental disorders is 15–18 years (Solmi et al., 2022). That is, some mental disorders originate during neurodevelopmental phases and peak in middle or late adolescence. In addition, another recent review showed that approximately 20–50% of adolescents present comorbidity of anxiety and a chronic physical illness, such as asthma or diabetes (Cobham et al., 2020), and other studies have shown that pediatric patients with chronic disease are at an increased risk of both anxiety and depressive symptoms (Ferro et al., 2016; Lacomba-Trejo et al., 2020; Arrondo et al., 2022; Määttä et al., 2022). In psychological and physical comorbidity, depression or anxiety is associated with a worse prognosis and adherence to treatment (Zheng et al., 2020) and has important psychosocial implications, such as decreased academic performance and relationships with peers (Pagerols et al., 2022). The treatment of chronic diseases during childhood and adolescence poses challenges for physical and mental health because they are periods of rapid growth, emotional instability and physiological changes accompanied by important socialization and individualization processes (Russo, 2022).

In this context, the salutogenic perspective emphasizes the so-called general resources of resistance, which are biological, material and psychosocial factors such as money, knowledge, experience, self-esteem, healthy habits, commitment, social support, cultural capital, intelligence, traditions and life vision, in the promotion of physical and mental health (Antonovsky, 1987; Braun-Lewensohn et al., 2017). From this perspective, if a person has these resources, he or she has more opportunities to face the challenges of life. This approach has been used in different studies in health psychology and in the child and adolescent population (Roehrich et al., 2021; Shorey and Nebby, 2021; Oh et al., 2023). However, more importantly, individuals must have the ability to use these resources, that is, have a sense of coherence, to maintain health and promote quality of life; since illness is perceived as a threat or stressor also in childhood and adolescence (Braun-Lewensohn et al., 2017). The sense of coherence refers to the ability to understand and integrate (comprehensibility), manage (manageability) and give meaning (significance) to diseases, reducing the perceived threat of chronic diseases and favoring their integration into personal identity. This indicates the importance of the perceived threat of diseases in the well-being of children and adolescents with chronic diseases (Leventhal et al., 2016); however, research on this construct is still scarce. Preliminary evidence suggests that a greater sense of coherence is related to adherence to treatment, self-care skills, health behaviors, perceived health, quality of life and general well-being, as well as the sense of self and identity in children and adolescents with chronic diseases (Apers et al., 2013; Aujoulat et al., 2017; Elissa et al., 2020). In general, in childhood and adolescence, males, younger individuals, individuals with greater cognitive functioning, nonimmigrants, nonsmokers and individuals with available support from peers and relatives have higher levels of coherence, which leads to better perceived health, better quality of life and more positive coping strategies (Shorey and Nebby, 2021).

From a salutogenic perspective, self-esteem is a psychological resource that children and adolescents can foster (Braun-Lewensohn et al., 2017). Specifically, in chronic diseases, self-esteem helps adolescents overcome the negative effects of exposure to risk and the perceived threat of diseases, being related to sense of coherence and feelings of control (Shorey and Nebby, 2021; Hards et al., 2023). Good self-esteem in children and adolescents facilitates emotional regulation, positive relationships with peers and resistance to social pressures, psychological well-being and positive thoughts about the future, helping to reduce internalizing symptoms (Peñate et al., 2020; Schoeps et al., 2021; Crocetti et al., 2022). However, according to an extensive meta-analysis, adolescents with chronic diseases may have worse self-esteem than do healthy adolescents (Pinquart, 2013); however, it has been suggested that related aspects, not diseases themselves, negatively influence self-esteem, e.g., uncontrolled asthma (Letitre et al., 2014), with inconsistent results for all chronic conditions, with adolescents with chronic pain having the worst self-esteem and those with asthma or diabetes having self-esteem scores similar to those for healthy adolescents (Hards et al., 2023). Regarding sex, adolescent boys tend to have better self-esteem than do girls, both healthy and with chronic disease (Lacomba-Trejo et al., 2018; Peñate et al., 2020; Hards et al., 2023). Specifically, girls with chronic diseases such as asthma, allergies or diabetes seem to have lower self-esteem (Pinquart, 2013; Lacomba-Trejo et al., 2018; Hards et al., 2023), less sense of coherence, greater perceived threat of diseases and lower perceived quality of life than do boys with similar conditions (Colombo et al., 2019; Elissa et al., 2020; James and Caballero, 2020), as well as a greater probability of developing depression (Vazquez-Ortiz et al., 2020). Regarding age, adolescence in pediatric patients with chronic disease is associated with the presence of anxiety-depressive symptoms (Vazquez-Ortiz et al., 2020); therefore, adolescence can be a factor that influences the presentation of emotional disorders. The early onset of chronic disease (before age 7) can predict a higher level of externalizing problems, and late onset predicts internalizing and thinking problems in adolescence (Määttä et al., 2022).

In addition to psychological or personal resources, the salutogenic perspective emphasizes the role of contextual factors, such as family members, in promoting health and preventing disease (Braun-Lewensohn et al., 2017). As reported in reviews and meta-analyses, a low level of conflict and high family cohesion are the most influential variables in the prevention of internalizing and externalizing problems (Leeman et al., 2016) as well as a better adherence to treatment (Psihogios et al., 2019). However, other variables such as affectionate and positive communication and family flexibility may also be relevant. For example, there is evidence that emotional support from parents functions as a protective factor in asthma, showing a greater sense of control over treatment and fewer emotional problems (Vazquez-Ortiz et al., 2020). Additionally, in pediatric asthmatic patients, self-esteem and the perceived threat of the disease has been shown to be a mediator between family factors and quality of life perceived by children and adolescents (Valero-Moreno et al., 2023). The aforementioned study indicated that favorable family environments were characterized by low psychological control (which indicates greater family flexibility) and high affective support, which functioned as facilitators in self-management and disease control and in the quality of life of the children and adolescents with asthma.

Despite great advances in the study of chronic pediatric disease in relation to emotional distress, most studies focus on a single disease and only one type of factor, i.e., personal or family. Therefore, there are few studies that include children and adolescents with different diseases, which can have similar psychological impacts with respect to general psychological resources and family factors. Taking into account the gaps in the current literature, the objective of this study was to analyze the joint capacity of demographic, personal and family variables to predict the emotional distress of children and adolescents with different chronic diseases. Specifically, the type of chronic disease (pneumoallergy or endocrine), the sociodemographic variables sex and age (12–16 years), the personal variables self-esteem and perceived threat of the disease (indicating a low sense of coherence) and the family variables affection, communication, and psychological and behavioral control were used to predict four types of emotional distress in adolescents, defined according to the combined presence of high or low levels of anxiety and depression. The hypotheses were as follows:

*First Hypothesis*: Girls and older adolescents with chronic diseases have higher levels of emotional distress than do boys and younger adolescents.

*Second Hypothesis*: Self-esteem and functional family characteristics (affection and communication) are negatively associated with emotional distress, and the perceived threat and dysfunctional family characteristics (psychological control and behavioral control) are positively associated with emotional distress.

*Third Hypothesis*: Older adolescent girls with low self-esteem, high perceived threat and dysfunctional family characteristics (low communication and high psychological and behavioral control), regardless of the type of disease, have higher levels of anxiety-depressive symptoms.

## 2 Materials and methods

### 2.1 Participants

The participants were 495 adolescents with pneumoallergy (asthma or allergy) and endocrinology disorders (short stature or diabetes mellitus type I, DM1) between 12 and 16 years of age treated at different hospitals in the Valencian region (*M* = 13.55; *SD* = 1.26), using a cross-sectional study design with incidental sampling. From the total sample, 61.60% (*n* = 305) of the participants were male, 60.80% had pneumoallergy disorders and 39.20% had different endocrinologic disorders: 17.30, 22.10, 8.40, 36.00, and 16.20% were adolescents with DM1, short stature, asthma, allergic asthma, and allergies, respectively. The time since diagnosis was approximately 6 years (*M* = 71.40 months; *SD* = 98.42; min = 6; max = 1728 months). By choosing a 99% confidence level and a sample size of 495 participants the margin of error would be ±4.6%, which means that the study has enough participants in order to have sufficient statistical power.

### 2.2 Instruments

Variables related to the type of disease, sex and age were assessed *ad hoc*.

The psychological variables were assessed using well-established self-report questionnaires, validated for Spanish adolescents. The reported reliability values are based on the current sample. Cronbach’s alpha values near 0.70 are considered acceptable (Taber, 2018).

#### 2.2.1 Emotional adjustment

The version of the Hospital Anxiety and Depression Scale (HADS) (Zigmond and Snaith, 1983) version for adolescents (Valero-Moreno et al., 2019) was used to assess anxious and depressive symptomatology. This version contains 11 items used to estimate symptomatology severity; the items are scored using a Likert scale from 0 to 3. The instrument has a minimum score of 0 and a maximum score of 33. Higher scores on this instrument mean higher levels of anxiety and depression. Both the original instrument and the version adapted for adolescents were found to have adequate reliability. In this study, the instrument showed an adequate reliability index (Cronbach’s alpha =0.74).

#### 2.2.2 Illness threat perception

The Brief Illness Perception Questionnaire (B-IPQ) (Broadbent et al., 2006) measures patients’ cognitive and emotional representations of their illness regarding perceived threat. In this study, a version adapted and validated for adolescents (9–16 years) (Valero-Moreno et al., 2020) was used. The instrument contains 5 items that are scored using a Likert scale from zero to 10, measuring cognitive and affective aspects of perceived threat, such as daily interference (“How much does your illness affect your life?”), emotional impact (“How does your illness affect you emotionally?”), and duration beliefs (“How long do you think your illness will last?”). The range of scores is 0 to 50, with higher scores being understood as a greater sense of threat of illness. The scale has previously shown good psychometric properties (Broadbent et al., 2006; Valero-Moreno et al., 2020); in the present study, reliability was adequate (Cronbach’s alpha =0.74).

#### 2.2.3 Self-esteem

A Spanish (and validated for adolescents) version of the Rosenberg Self-Esteem Scale (RSE; Rosenberg, 1965) was used (Atienza et al., 2000). The scale consists of 10 items focusing on feelings of self-respect and self-acceptance; the items are scored using a Likert scale from 1 (strongly disagree) to 4 (strongly agree). The range of scores is from 10 with the minimum to 40 with the maximum, higher scores on this questionnaire are understood as higher self-esteem in the original study, the reliability index was 0.92 (Atienza et al., 2000). In the present study, the reliability was adequate (Cronbach’s alpha =0.79).

#### 2.2.4 Family styles

The Parental Styles questionnaire (Oliva et al., 2007, 2008) was created to evaluate adolescents’ perception of their parents’ parenting style; items are scored using a Likert scale from 1 (strongly disagree) to 6 (strongly agree). The original scale consists of 41 items assessing six dimensions. For the purposes of the study, only three dimensions were used: affect and communication, which refers to perceived emotional support from parents (“I feel supported and understood,” “Shows interest in me when I am sad and angry”), correlates with adolescent adjustment indices and is characteristic of a democratic style. The scale of affection and communication is composed of eigth items, with a minimum score of 8 and a maximum score of 48. A higher score means a greater perception by the adolescent that his or her parents promote affection and communication within the family. Behavioral control, which refers to perceived control over the activities and people in the respondent’s life, as well as the establishment of limits (“Tries to know where I go when I go out,” “Tries to know what I do in my free time”) and correlates with adolescent adjustment indices unless associated with psychological control. The behavioral control dimension is composed of six items, with a minimum score of 6 and a maximum score of 36. A higher score means that parents exercise greater behavioral control by imposing a greater number of limits and rules. And psychological control, which refers to intrusive and manipulative strategies, such as the induction of guilt or the withdrawal of affection, used by parents when the adolescent chooses behavior that they do not approve of (“Tries to continuously control my way of being and thinking,” “They make me feel guilty when I do not do what they want”), correlates with indices of adolescent maladjustment and is characteristic of an authoritarian style. The psychological control dimension is composed of eigth items, with a minimum score of 8 and a maximum of 48. A higher score indicates that parents exert greater psychological control through emotional blackmail. The original scale showed adequate reliability indices (Oliva et al., 2008). In the present study, the 3 subscales showed adequate reliability indices (Cronbach’s alpha =0.74–0.81).

### 2.3 Procedure

A convenience sample was obtained from three hospitals in the Valencian region. The adolescents completed the questionnaires after legal guardians or parents signed an informed consent form. Incomplete questionnaires were removed and not analyzed. The procedure consisted of identifying all adolescents with chronic diseases who attended consultations at the hospital. The inclusion criteria were an age between 12 and 16 years and presenting pneumoallergy (asthma or allergy) or endocrinology disorders (short stature or diabetes mellitus type I, DM1) for at least 6 months. The work complies with the ethical criteria of the Helsinki Declaration (2013) and has been endorsed by the Ethics Committee of the corresponding institutions (UV-INV_ETICA-1226194).

### 2.4 Data analysis/statistics

First, typologies were created by combining two indicators (anxiety and depression) with two different levels (low and high). For anxiety and depression, high levels were determined by selecting scores above 60th percentile and low levels were determined by selecting scores below the 40th percentile, thus creating high risk and low risk groups. This classification served to remove mean values and to polarize low and high levels. In this procedure, a total of 38 were eliminated and 461 participants were included in the typologies. In this way, four typologies were formed. Typology I included adolescents with high levels in both dimensions. Typology II included adolescents with high anxiety and low levels of depression. Typology III included adolescents with low levels of anxiety and high levels of depression. Finally, Typology IV included adolescents with low levels of anxious-depressive symptomology. Before conducting statistical analyses, we tested for assumptions of normality, multicollinearity, homoscedasticity, and independent errors, obtaining satisfactory results. Next, comparisons of means were performed for all study variables by typology using one-factor ANOVA, and contingency tables were generated for the sociodemographic variables. In addition, 3-step logistic regression was carried out to predict the four typologies. Type of chronic disease, sex and age were introduced in the first step, self-esteem and perceived threat were introduced in the second step, and communication and psychological and behavioral control were introduced in the third step. Finally, logistic regression was carried out to determine the factors relevant to changes from a risk typology (Typology I, II, and III) to a non-risk typology (Typology IV, with low levels of anxiety and depression). In this way, the factors required to decrease emotional distress risk in adolescents with chronic disease were identified.

## 3 Results

### 3.1 Typologies of emotional distress (anxiety and depression): mean comparisons

Table 1 presents descriptive statistics for all studied variables (of the participants included in the study). A total of 36.44% (*n* = 168) of the participants were classified as typology I (high anxiety and depression), 14.75% (*n* = 68) as typology II (high anxiety and low depression), 17.14% (*n* = 79) as typology III (low anxiety and high depression) and 31.67% (*n* = 146) as typology IV (low anxiety and depression). The following criteria was used to differentiate high and low levels of anxiety and depression: scores above the 60th percentile indicated high levels of anxiety and depression, and scores below the 40th percentile indicated low levels of anxiety and depression.

**Table 1:** Descriptive statistics of all participants included in the study.

	*n* (%)	M (SD)	Min	Max
Type of chronic disease				
Pneumoallergic problems	301 (60.80)			
Endocrinologic problems	194 (39.20)			
Gender				
Boys	305 (61.62)			
Girls	190 (38.38)			
Age				
12 years	131 (26.46)			
13 years	117 (23.64)			
14 years	130 (26.26)			
15 years	73 (14.75)			
16 years	42 (8.48)			
Self-esteem		32.92 (4.39)	12	40
Perception of threat		18.49 (9.65)	0	46
Affect and communication		41.85 (6.76)	16	107
Psychological control		20.90 (8.32)	8	47
Behavioral control		28.45 (5.95)	6	38
Anxiety		5.03 (3.22)	0	16
Depression		2.22 (2.19)	0	11

When comparing the means of all the study variables by typology (Table 2), using contingency or chi-square tables, differences were found in the typologies based on the type of chronic disease (*χ*^2^ = 8.51; *p* = 0.037), finding that adolescents with an endocrinological disease tended to be classified as typology IV, and those with pneumoallergy disorders tended to be classified as typology I. No gender differences were observed in each of the typologies created. There was an equal distribution between boys and girls in the four typologies. However, there was a higher percentage of females than males classified as typology I and a higher percentage of males than females classified as typology IV (*χ*^2^ = 6.59; *p* = 0.086). When differentiating between the four typologies according to age group, no differences were found either. Again, a similar distribution was observed in the typologies based on age groups. However, as seen in the Table 2, older age groups (15 or 16 years) were more concentrated in typology I (*χ*^2^ = 15.99; *p* = 0.191).

**Table 2:** Mean comparisons between the four typologies (combinations of high/low levels of anxiety and depression).

Variables	Group	Typology I: High anxiety & High depression	Typology II: High anxiety & Low depression	Typology III: Low anxiety & High depression	Typology IV: Low anxiety & Low depression	*χ*^2^ test			
		*n* (%)	*n* (%)	*n* (%)	*n* (%)	*χ*^2^	*p*	V	
Type of chronic disease	Pneumoallergic	100 (39.70)	39 (15.50)	44 (17.50)	69 (27.40)	8.51	0.037	0.144	
	Endocrinological	47 (29.90)	26 (16.60)	21 (13.40)	63 (40.1)				
Gender	Female	75 (41.0)	32 (17.50)	26 (14.20)	50 (27.30)	6.59	0.086	0.120	
	Male	93 (33.50)	36 (12.90)	53 (19.10)	96 (34.50)				
Age	12 years	38 (32.50)	19 (16.20)	19 (16.20)	41 (35.0)	15.99	0.191	0.108	
	13 years	36 (32.70)	16 (14.50)	23 (20.90)	35 (31.80)				
	14 years	42 (33.60)	18 8 (14.40)	19 (15.20)	46 (36.80)				
	15 years	29 (42.0)	8 (11.60)	14 (20.30)	18 (26.10)				
	16 years	23 (57.50)	7 (17.50)	4 (10.0)	6 (15.0)				
						One-way *ANOVA*			
		*M (SD)*	*M (SD)*	*M (SD)*	*M (SD)*	*F*	*p*	*η^2^*	*Post-hoc Bonferroni*
Self-esteem		31.34 (4.43)	34.20 (4.50)	32.97 (3.67)	34.09 (4.16)	12.81	0.001	0.082	I < II I < III I < IV
Perception of threat		21.01 (10.30)	17.39 (9.07)	18.38 (9.23)	16.26 (8.58)	6.32	0.001	0.044	I < IV
Affect and communication		40.29 (6.83)	42.57 (5.52)	42.66 (9.67)	42.94 (4.91)	4.92	0.002	0.032	I < III I < IV
Psychological control		23.70 (8.92)	19.03 (7.65)	20.87 (8.24)	18.15 (6.55)	13.78	0.001	0.085	I < II I > IV
Behavioral control		29.44 (5.37)	27.89 (5.94)	28.51 (5.11)	27.54 (6.63)	2.99	0.031	0.020	I > IV

M, Mean; SD, Standard deviation; F, F-value; *p*, *p*-value of significance; *η*^2^ eta, effect size. ^*^p ≤ 0.05, *χ*^2^ = chi-square value, V, the size of Crammer’s V effect.

One-way ANOVA was used to analyze the rest of the variables by typology. There were differences for self-esteem, i.e., differences between typology I (risk) and the other typologies, with participants in the former group having lower self-esteem scores (*F* = 12.81, *p* =. 001, *η*^2^ = 0.082). Regarding perceived threat of the disease, there were differences between typology I (risk typology) and typology IV (healthy typology), with the participants in the former group having higher mean scores for perceived threat (*F* = 6.32, *p* =. 001, *η*^2^ = 0.044). Finally, regarding parenting styles, there were differences among typologies for all variables. For the affection and communication dimension, participants in the typology I group had lower scores than those in the typology III and typology IV groups. Differences were observed in psychological control between typologies I and II, as well as between typologies II and IV. These findings once again highlight that participant in typology I exhibited higher scores of psychological control. Furthermore, when it comes to behavioral control, participants in typology I outscored those in typology IV.

### 3.2 Typologies of emotional distress (anxiety and depression): logistic regression

Table 3 summarizes the predictive models for each typology. For typology I (high anxiety and high depression), all three models were statistically significant, explaining 5, 12 and 26% of the variance in belonging to typology I (Table 3). Age, perceived threat, and psychological and behavioral control were positive predictors, and self-esteem was a negative predictor in the final model (step three). For typologies II (high anxiety and low depression) and III (low anxiety and high depression), the predictive models did not reach significance, and no predictive capacity was observed for any variable. Finally, the predictive models for typology IV (low anxiety and low depression) were significant, explaining 6, 8, and 17% of the variance. The significant predictors were age and psychological and behavioral control, all with negative beta coefficients.

**Table 3:** Logistic regressions for typologies.

Variable	Typology I: High anxiety & High depression (0 = No, 1 = Yes)			Typology II: High anxiety & Low depression (0 = No, 1 = Yes)			Typology III: Low anxiety & High depression (0 = No, 1 = Yes)			Typology IV: Low anxiety & Low depression (0 = No, 1 = Yes)		
	B	SE	Wald	B	SE	Wald	B	SE	Wald	B	SE	Wald
Step 1												
Type of chronic disease	−0.29	0.56	1.26	0.23	0.33	0.49	−0.20	0.34	0.33	0.30	0.26	1.35
Gender	0.38	0.24	2.57	0.36	0.31	1.37	−0.51	0.33	2.33	−0.36	0.26	1.98
Age	0.27	0.09	8.72^**^	0.01	0.12	0.01	−0.02	0.12	0.02	−0.32	0.10	9.65^**^
NR^2^	0.05^**^			0.01			0.01			0.06^**^		
Step 2												
Type of chronic disease	−0.18	0.27	0.44	0.12	0.34	0.12	−0.23	0.35	0.45	0.23	0.27	0.75
Gender	0.18	0.25	0.53	0.48	0.32	2.15	−0.45	0.34	1.75	−0.24	0.27	0.79
Age	0.24	0.10	6.63^**^	0.03	0.13	0.06	−0.01	0.12	0.00	−0.30	0.10	8.32^**^
Self-esteem	−0.08	0.03	7.39^**^	0.07	0.04	3.04	0.02	0.04	0.31	0.03	0.03	1.08
Perception of threat	0.04	0.01	8.73^**^	−0.01	0.02	0.49	−0.01	0.02	0.33	−0.03	0.01	4.47^*^
NR^2^	0.12^***^			0.03			0.02			0.08^***^		
Step 3												
Type of chronic disease	−0.13	0.28	0.22	0.11	0.34	0.11	−0.21	0.35	0.37	0.24	0.28	0.78
Gender	0.36	0.27	1.77	0.36	0.33	1.16	−0.45	0.35	1.68	−0.38	0.28	1.80
Age	0.29	0.10	7.75^**^	0.03	0.13	0.07	0.02	0.12	0.02	−0.34	0.11	9.67^**^
Self-esteem	−0.10	0.03	10.10^***^	0.07	0.04	2.79	0.02	0.04	0.33	0.04	0.03	1.78
Perception of threat	0.03	0.01	4.40^*^	−0.01	0.02	0.12	−0.01	0.02	0.27	−0.02	0.01	2.42
Affect and communication	−0.04	0.02	2.16	0.00	0.03	0.00	0.04	0.03	1.53	0.02	0.03	0.31
Psychological control	0.06	0.02	10.42^***^	−0.04	0.02	3.07	0.01	0.02	0.16	−0.05	0.02	5.90^*^
Behavioral control	0.09	0.03	12.93^***^	−0.02	0.03	0.41	−0.01	0.03	0.06	−0.07	0.02	9.35^**^
NR^2^	0.26^***^			0.05			0.03			0.17^***^		

Type of chronic disease: 1 = pneumoallergic problems, 2 = endocrinologic problems; Gender: 1 = boys, 2 = girls; B = regression coefficient. SE = Standard Error. NR^2^ = Nagelkerke R^2^. ^*^p ≤ 0.05. ^**^p ≤ 0.01. ^***^p ≤ 0.001.

### 3.3 Differences between risk and non-risk typologies: logistic regression

We further analyzed which of the studied variables (type of chronic disease, sex, age, self-esteem, perceived threat, affect, communication, and psychological and behavioral control) predicted belonging to a risk typology (typologies I, II and III) or to the non-risk typology (typology IV) (Table 4). There were significant differences between belonging to the high-risk typology I group (high anxiety & high depression) and the non-risk typology IV group (low anxiety and low depression). Thus, the results suggest that increasing self-esteem and reducing psychological and behavioral control, as well as younger age, increase the probability of belonging to the non-risk typology IV group rather than the high-risk typology I group (high anxiety and high depression), explaining 34% of the variance in the difference between the two typologies.

**Table 4:** Logistic regressions for the difference between risk typologies (I, II, III) and non-risk typology (IV).

Variables	Typology I Typology IV (1 = risk, 0 = non-risk)			Typology II Typology IV (1 = risk, 0 = non-risk)			Typology III Typology IV (1 = risk, 0 = non-risk)		
	B	SE	Wald	B	SE	Wald	B	SE	Wald
Step 1									
Type of chronic disease	−0.36	0.30	1.39	−0.04	0.37	0.01	−0.35	0.38	0.83
Gender	0.41	0.29	2.06	0.60	0.36	2.78	−0.14	0.38	0.13
Age	0.37	0.11	10.81^***^	0.26	0.15	3.08	0.24	0.16	2.44
NR^2^	0.09^***^			0.05			0.03		
Step 2									
Type of chronic disease	−0.24	0.31	0.58	−0.09	0.38	0.06	−0.37	0.39	0.93
Gender	0.22	0.30	0.51	0.60	0.37	2.60	−0.19	0.39	0.24
Age	0.33	0.11	8.06^**^	0.27	0.15	3.07	0.23	0.16	2.07
Self-esteem	−0.07	0.04	3.38	0.04	0.04	0.69	−0.00	0.04	0.00
Perception of threat	0.04	0.02	6.70^**^	0.01	0.02	0.25	0.01	0.02	0.50
NR^2^	0.16^***^			0.06			0.04		
Step 3									
Type of chronic disease	−0.35	0.34	1.11	−0.10	0.38	0.07	−0.40	0.40	1.00
Gender	0.44	0.34	1.67	0.61	0.39	2.44	−0.09	0.41	0.05
Age	0.40	0.13	9.28^**^	0.29	0.16	3.32	0.30	0.17	3.26
Self-esteem	−0.12	0.04	8.66^**^	0.03	0.04	0.52	−0.01	0.05	0.09
Perception of threat	0.02	0.02	1.81	0.01	0.02	0.21	0.01	0.02	0.34
Affect and communication	−0.03	0.03	1.17	−0.00	0.04	0.00	0.01	0.04	0.10
Psychological control	0.07	0.02	9.71^**^	0.01	0.03	0.14	0.05	0.03	3.24
Behavioral control	0.12	0.03	13.74^***^	0.03	0.03	0.85	0.06	0.03	2.77
NR^2^	0.34^***^			0.07			0.10		

Type of chronic disease: 1 = pneumoallergic problems, 2 = endocrinologic problems; Gender: 1 = boys, 2 = girls; B = regression coefficient. SE = Standard Error. NR^2^ = Nagelkerke *R*^2^. ^*^p ≤ 0.05. ^**^p ≤ 0.01. ^***^p ≤.

## 4 Discussion

The objective of this study, framed in a salutogenic perspective, was to analyze the joint role of demographic, personal and family factors in the emotional distress of adolescents with different types of chronic diseases to better understand the mechanisms of physical and psychological comorbidity in this vital stage and thus optimize possible interventions with this population and their families. The results of the study show that the relevant variables for belonging to typologies at higher risk of emotional distress were self-esteem and family variables.

The study of emotional distress in adolescents with chronic diseases becomes an intriguing challenge for the development of optimal interventions that support the cultivation of healthy life projects, given the characteristics of adolescence, the presence of physical and psychological comorbidities, and the combined influence of these factors on vital development. This is evident in the works of Arrondo et al. (2022); Cobham et al. (2020); Ferro et al. (2016); Lacomba-Trejo et al. (2020); Määttä et al. (2022); Pagerols et al. (2022); and Zheng et al. (2020). The salutogenic perspective (Antonovsky, 1987; Braun-Lewensohn et al., 2017) focuses on general psychological resources such as self-esteem and contextual factors such as family members that make significant life experiences and greater resilience against disease possible (Roehrich et al., 2021; Shorey and Nebby, 2021; Oh et al., 2023), of which this study provides evidence. In general, the results are consistent with the theory (Hypothesis 2); that is, self-esteem serves as a positive personal resource, reducing emotional distress, and perceived threat of the disease predicts greater discomfort. Likewise, the emotional distress of adolescents with chronic illness is consistently related to functional family factors (affect and communication decrease distress) and dysfunctional factors (psychological and behavioral control increase distress). However, the aim of this study was to determine the joint influence of these factors, as well as sex, age and type of chronic disease (hypotheses one and three). In this sense, hypotheses one and three can only be partially accepted.

Regarding the first hypothesis, sex and age did not consistently predict emotional distress in adolescent patients. Previous studies have observed that sex is differential in variables such as self-esteem, both in healthy and chronically ill adolescents (Lacomba-Trejo et al., 2018; Peñate et al., 2020; Hards et al., 2023), both in adolescents with asthma and allergies or diabetes (Pinquart, 2013; Lacomba-Trejo et al., 2018; Hards et al., 2023), in the sense of coherence, perceived threat of the disease and quality of life (Colombo et al., 2019; Elissa et al., 2020; James and Caballero, 2020) and in the risk of presenting anxiety-depressive symptoms in pediatric patients with chronic disease (Vazquez-Ortiz et al., 2020). However, in this study, there were no sex differences in the probability of different types of risk of emotional distress, and in the regression analyses, only age showed a predictive capacity. That sex was not a relevant variable may be due to different issues, from the sample size to the inclusion of different types of disease (which could have differential impacts based on sex). Age, on the other hand, consistently predicted greater emotional distress, an effect that was maintained when the other study variables were incorporated into the model. Thus, late adolescence (16 years) seems to increase the risk of emotional distress in pediatric patients with chronic disease, regardless of the type of disease and personal and family resources.

Regarding the third hypothesis, the joint influence of the study variables and dysfunctional family factors (psychological and behavioral control) showed a clear and consistent effect on the emotional distress of adolescents, and family affection lost its individual effect when other family variables were included, with personal variables (self-esteem and perceived threat) having inconsistent effects. Participants in the typology I group, with high emotional distress, had worse self-esteem and greater perceived threat; however, when combining variables, both lost relevance in the prediction of emotional distress. More specifically, self-esteem had predictive power for high emotional distress (typology I) but not for moderate (typologies II and III) or low (typology IV) emotional distress, and perceived threat lost its explanatory power for adolescent emotional distress when family factors were included in the model. The outcomes regarding self-esteem in this study may be influenced by the specific diseases considered (pneumoallergy and endocrine disorders), suggesting that these conditions might not impact self-esteem to the same extent as other chronic health, as observed by Hards et al. (2023), comparing the self-esteem of sick and healthy children and adolescents. Likewise, it would be interesting to investigate in greater depth the relationship between adolescents’ perceived threat of the disease and greater psychological and behavioral control by parents. It is possible that personal salutogenic factors play mediating roles over family factors, as evidenced by a previous study on the perceived quality of life of adolescent children with asthma (Valero-Moreno et al., 2023), or that the activation of personal resources can only occur in this vital stage if favorable contextual factors (family in this case) are present (Roehrich et al., 2021; Shorey and Nebby, 2021; Oh et al., 2023). Future research could further explore this relationship to achieve a greater understanding of the role of contextual factors in the activation of personal psychological resources of salutogenesis in the chronically ill pediatric population and, more specifically, in adolescence. Finally, the type of chronic disease, as postulated in the third hypothesis, did not show relevance in the prediction of emotional distress when the other study variables, that is, psychological resources and relatives, are taken into account. However, the initial comparison of means showed that adolescents with chronic pneumoallergy disorders presented greater anxiety-depressive symptoms than did patients with endocrine disorders. This suggests the usefulness of investigating populations that include different types of diseases to obtain generalizable results regarding the role of chronic diseases in adolescence.

This study has some limitations that must be taken into account, for example, the sample size or the self-reported nature of the data. However, the results provide evidence of the importance of age—or the adolescent stage itself—the perceived threat of the disease and family factors related to flexibility (psychological and behavioral control) in the emotional distress of adolescents with chronic diseases. Likewise, the results suggest the need to study in greater depth the complex role of sex and self-esteem as well as to further differentiate results by different types of chronic disease.

## 5 Conclusion

In conclusion, the study highlights age, psychological control, and behavioral control as key differentiating factors between individuals at risk and those not at risk of emotional distress. The findings emphasize the potentials mediating or modulating roles of sex, self-esteem, and perceived threat in these relationships. This suggests a practical implication for family interventions in this population, emphasizing the importance of promoting family flexibility in nurturing relationships. The theoretical contribution underscores the complexity of relationships among these factors, suggesting that the salutogenic perspective’s relationships are not simply summative or cumulative. Further research is needed to explore mediating or moderating relationships while controlling for disease type and considering the influence of sex. Additionally, a comprehensive comparative analysis that includes various chronic conditions would contribute to a more thorough understanding of these dynamics.

## Data availability statement

The data that support the findings of this study are available from the corresponding author, SV-M, upon reasonable request.

## Ethics statement

The studies involving humans were approved by (UV-INV_ETICA-1226194)-Universitat de Valencia. The studies were conducted in accordance with the local legislation and institutional requirements. Written informed consent for participation in this study was provided by the participants’ legal guardians/next of kin.

## Author contributions

SP-Z: Funding acquisition, Methodology, Writing – original draft, Writing – review & editing. KS: Formal analysis, Funding acquisition, Methodology, Writing – original draft. MP-M: Conceptualization, Supervision. LL-T: Investigation, Data curation. SV-M: Funding acquisition, Investigation, Methodology, Project administration, Writing – original draft, Writing – review & editing.
